# High-Sensitivity Solidly Mounted Resonator Load Sensor Based on AlN/AlScN Heterostructure

**DOI:** 10.3390/s25237288

**Published:** 2025-11-29

**Authors:** Wanqing Zuo, Xiyu Gu, Tingting Yang, Qinwen Xu, Haiyang Li, Yao Cai, Chengliang Sun

**Affiliations:** 1 Department of Electrical and Electronic Engineering, The Hong Kong Polytechnic University, Hong Kong, China; 2 Hubei Key Laboratory of Electronic Manufacturing and Packaging Integration, School of Integrated Circuits, Wuhan University, Wuhan 430072, China; 3 The Institute of Technological Sciences, Wuhan University, Wuhan 430072, China

**Keywords:** piezoelectric thin film, solidly mounted resonator (SMR), AlN/AlScN composite film, load sensor

## Abstract

Bulk acoustic wave (BAW) resonators, with their exceptional high-frequency performance and excellent quality factor, have become a key driver of advances in sensing technology. This study reports the fabrication and characterization of a force sensor based on a solid mounted resonator (SMR) structure. This SMR device utilizes a high resonance frequency of 2.257 GHz as its core sensing element. The operational mechanism involves the application of an external load inducing localized downward mechanical deformation in the SMR film at the pin contact region, thereby generating significant in-plane compressive stress within the piezoelectric layer. The applied strain modifies the intrinsic elastic and piezoelectric constants of the film, thereby changing both the acoustic phase velocity and the electromechanical coupling coefficient ($K_{t}^{2}$), which ultimately leads to a measurable shift in the resonance frequency. The experimental results reveal a deterministic and robust correlation between the resonance frequency shift and the applied load, which forms a precise function relationship enabling the device to achieve a high sensitivity of 37.79 MHz/N. This indicates that it may possess good application and development potential in various complex industrial fields.

## 1. Introduction

In recent years, precision force sensors based on piezoelectric resonators have attracted widespread attention in various fields, including microrobotics, biomedical diagnostics, structural health monitoring, and advanced manufacturing. Force sensors are a type of pressure sensor that can convert tensile and compressive forces into corresponding electrical signals [1,2]. While Al-Dahiree et al. demonstrated structural optimization of strain gauge load cells through finite element analysis, Diah et al. concurrently provided a systematic assessment of their industrial integration, with both studies identifying limitations in the sensitivity, temperature stability, and electromagnetic interference resistance [3,4]. Kim et al. systematically investigated the use of traditional piezoelectric sensors for static force measurement, yet their analysis identified the fundamental challenge of charge leakage that inherently limits measurement stability under sustained loads [5]. Mishra et al. conducted a comprehensive analysis of the design, materials, and applications of flexible capacitive pressure sensors. Although it is stable and easy to integrate, it is vulnerable to electromagnetic interference and parasitic capacitance, which will cause loss of signal integrity and low sensitivity in the industrial environment full of noise [6]. Resonant sensors address these limitations by detecting variations in performance parameters induced by applied loads. These parameters include resonance frequency, quality factor, and amplitude. This approach enables direct digital output, offers superior stability and ultra-high sensitivity, and facilitates precise measurement of both static and dynamic forces.

Piezoelectric resonators have precise frequency control capabilities [7]. This is because they are micro devices manufactured using microelectromechanical systems (MEMS) technology and have unique advantages when used as high-precision resonant sensors. For example, they are small in size, compatible with complementary metal oxide semiconductor (CMOS) processes, highly sensitive, and have low manufacturing and testing costs [8,9,10]. Currently, piezoelectric resonators used for sensing are mainly divided into two categories: surface acoustic wave (SAW) resonators and bulk acoustic wave (BAW) resonators [11,12]. This study mainly uses BAW resonators. Among the piezoelectric materials used for BAW resonators, aluminum nitride (AlN) has become the mainstream choice due to its high acoustic velocity (about 11,000 m/s), low dielectric loss (tanδ < 0.1%), and CMOS process compatibility. However, the relatively low electromechanical coupling coefficient ($K_{t}^{2})$, specifically the thickness-mode electromechanical coupling coefficient, of pure AlN fundamentally limits its sensitivity. To overcome this limitation, this study employs Al_0.8_Sc_0.2_N alloy as the active piezoelectric layer, where scandium (Sc) doping significantly enhances the $K_{t}^{2}$ to ~15% [13]. This theoretically boosted the sensitivity by 2–3 times while maintaining high mechanical rigidity and structural integrity [14]. This combination of enhanced piezoelectric response and maintained mechanical rigidity lays the material foundation for achieving overall ultra-high sensitivity.

BAW resonators are finding increasing application due to their superior resonance frequency and electromechanical coupling coefficient, properties that enable higher sensitivity in sensing applications. Meanwhile, the solidly mounted resonator (SMR) demonstrates superior performance in force sensing applications due to its exceptional mechanical stability (stress concentration factor of 1.2–1.5), thermal stability (thermal resistance <20 K/W), and high-pressure tolerance (>500 MPa). This technology achieves remarkable specifications including ultra-high sensitivity (37.79 MHz/N), wide dynamic range (0.1–100 N), and compact footprint, outperforming conventional strain gauges, MEMS, and capacitive force sensors.

The utilization of BAW resonators for load sensing presents a promising alternative to traditional strain gauge-based approaches. Although BAW resonators have been extensively studied for applications in mass and pressure sensing, their direct implementation in load sensing remains largely unexplored. Nevertheless, the underlying sensing mechanisms share significant similarities, providing valuable insights for adaptation. For instance, Yan et al. demonstrated a SMR-based load sensor with a high sensitivity of 8–10 kHz·cm^2^·ng^−1^ at 3–4 GHz [15]. Similarly, Lin et al. developed a 2442.188 MHz film bulk acoustic resonator (FBAR) with a mass sensitivity of 3654 Hz·cm^2^·ng^−1^ via MEMS processing [16]. García-Gancedo et al. reported a gravimetric biosensor for protein detection with a sensitivity of 0.25 MHz·cm^2^·ng^−1^ based on an SMR platform [17]. Additionally, Xuan et al. explored pressure sensing using an FBAR with a reported sensitivity of approximately 1.5 ppm/kPa [18]. These pressure sensors operate by applying uniformly distributed loads to the resonator surface. Building upon these prior studies, it is reasonable to extend the BAW resonator approach to load sensing by applying localized or concentrated forces directly to the resonator, thereby enabling the realization of compact, highly sensitive load cells [19].

This work details the fabrication and systematic characterization of a high-sensitivity load sensor based on a scandium-doped aluminum nitride (AlScN) heterostructure within a solidly mounted resonator (SMR). While BAW resonators have been extensively explored for mass and pressure sensing, their application in direct load sensing under localized mechanical contact remains a developing area [20,21]. It is crucial to note that the transduction mechanism for load sensing fundamentally differs from the gravimetric principle of mass sensors; instead, it relies on the stress-induced modification of the piezoelectric film’s properties. Herein, we demonstrate that the AlN/AlScN composite piezoelectric layer serves as a highly effective sensing core, enabling the SMR to achieve an average force sensitivity of 37.79 MHz/N (16,744 ppm/N). The observed quadratic correlation between resonance frequency shift and applied load provides a stable and quantifiable metric for force transduction. These findings affirm the potential of AlScN-based SMRs as a promising technological route for accurate load detection in applications such as micro-robotics and advanced manufacturing systems.

## 2. Materials and Methods

**Figure 1:**
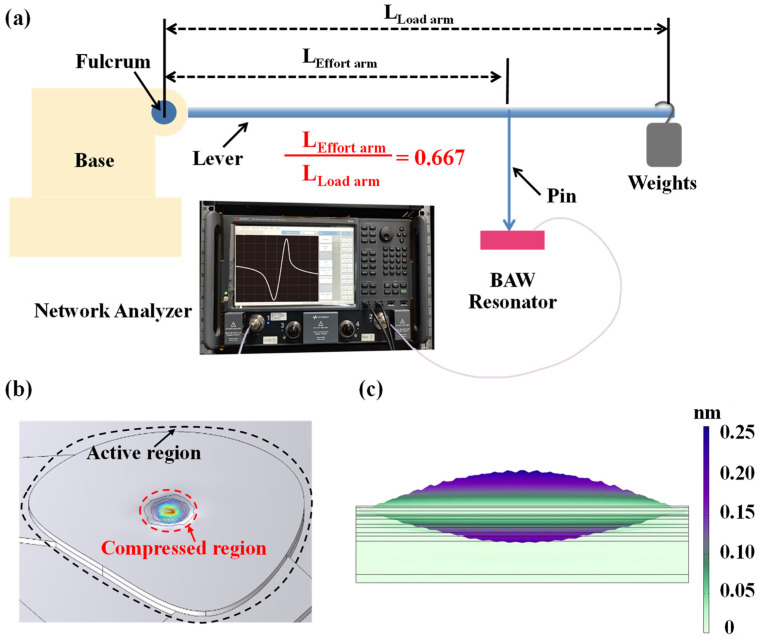
Experimental setup for load characterization of the BAW resonator. (**a**) Schematic of the lever-based load measurement system. (**b**) Simulated SMR deformation under pin force. (**c**) Simulated displacement field of the SMR’s fundamental resonant mode.

Based on the frequency sensitivity of SMR, we used a setup illustrated in Figure 1a to measure the load on SMR. The load measurement setup comprises four core components: a mechanical assembly, a series of applied masses, the test specimen, and associated testing instrumentation. The mechanical loading system comprises a base substrate and a lever mechanism with a contact pin, which transmits and concentrates the applied force at the central region of the SMR device.

The lever is connected to the base. According to the lever principle, weights generate graded gravitational forces as input, which are scaled by the ratio of the effort arm length to the load arm length (L1/L2) and applied as output forces to the SMR. The small contact area between the pin and the resonator enhances the contact surface stress, thereby amplifying the force-induced effect on the SMR’s phase velocity and improving device sensitivity. In this experiment, according to the specific device in the laboratory, the lever ratio L1/L2 is set to 0.667 (the actual arm length is L1 = 20 cm and L2 = 30 cm) to facilitate the application of the calibration force range suitable for characterizing the sensitivity of the sensor.

**Figure 2:**
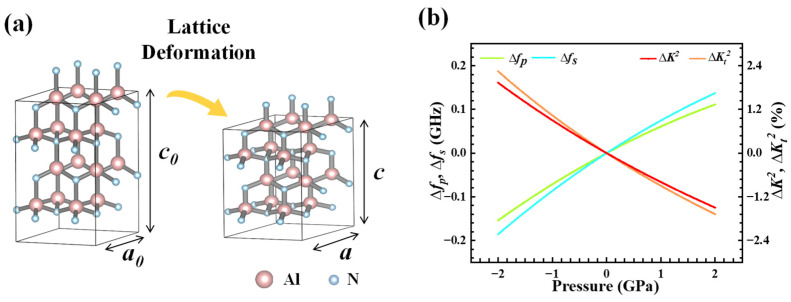
Effect of applied pressure on wurtzite AlN in an SMR structure. (**a**) Schematic of wurtzite structure hexagonal supercell variation in AlN when load applies at SMR. (**b**) Theoretical variation in resonance frequency and electromechanical coupling coefficient for an AlN-BAW resonator under stress.

Mechanistically, the application of external force induces downward deflection of the SMR film, as shown in Figure 1b. In this setup, SMR serves as the fundamental sensing unit, featuring a multilayered architecture composed of a piezoelectric layer (AlN/AlScN), metal electrodes (Mo), and a Bragg reflector (alternating Mo/SiO_2_ structure). Figure 1c illustrates the simulated vibration mode of the SMR. The acoustic energy is primarily confined within the piezoelectric layer, while the Bragg reflector effectively suppresses downward acoustic leakage into the substrate, confirming efficient acoustic confinement and standing-wave formation along the thickness direction. The deformation behavior of the internal piezoelectric film under applied external load is shown in Figure 2a. This microscopic visualization is the basis for understanding the macroscopic changes that occur in Figure 2b later. When a mechanical load is applied via the contact pin, it induces localized compressive stress within the piezoelectric film. At the atomic level, this stress manifests as a distortion of the wurtzite lattice, along the *c*-axis, leading to variations in the lattice constants (*a* and *c*). These structural changes directly alter the intrinsic material properties, including the polarization (piezoelectric response) and the elastic stiffness coefficients. The increase in elastic tensor and stiffness coefficient enhances the acoustic impedance of the material, thereby increasing the speed of sound waves and correspondingly raising the resonance frequency. These microscopic changes within these structures form the fundamental principle of our sensor’s high sensitivity characteristics, explain the corresponding changes in macroscopic parameters of the sensor. To clarify how such lattice deformation affects the device performance, Figure 2b presents the pressure-dependent evolution of the key parameters of AlN-based BAW resonators. This study employs first-principles and density functional theory (DFT) calculations to determine the material parameters, which are then incorporated into a BAW resonator frequency model to analyze the relationship between resonance frequency and external conditions. It should be noted that in continuum mechanics and materials science, there is a standard convention for defining Negative Stress (−σ) as Compressed Stress and Positive Stress (+σ) as Tensile Stress. However, the *x*-axis label in our Figure 2b is pressure. In this specific context, pressure is defined as a positive value for compression and a negative value for tension, which is the inverse of the stress (σ) convention. Therefore, in our research, the *x*-axis represents applied pressure, with negative values denoting tension and positive values denoting compression. The left *y*-axis shows the change in resonance frequency, and the right *y*-axis shows the change in the electromechanical coupling coefficient, both relative to their zero-stress values. This model confirms that compressive stress increases the resonance frequencies (*f_p_* and *f_s_*) and reduces the electromechanical coupling coefficients ($K^{2}$ and $K_{t}^{2}$), while tensile stress decreases the resonance frequencies (*f_p_* and *f_s_*) and increases the electromechanical coupling coefficients ($K^{2}$ and $K_{t}^{2}$). As this study applies load pressure through a pin tip, only the relevant parameter changes caused by compressive stress on the right half of the *x*-axis are discussed. Although the model in Figure 2b corresponds to an ideal single-layer piezoelectric film rather than the multilayer SMR structure used in this work, the same stress–piezoelectric coupling relationship governs the sensing principle of the present device.

**Figure 3:**
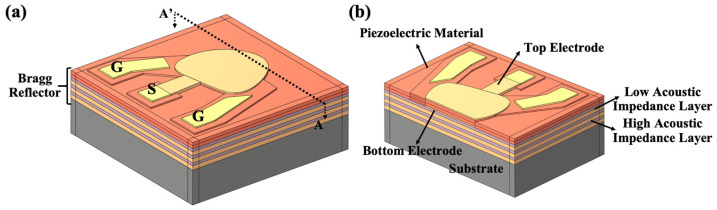
Schematic illustration of the SMR. (**a**) Three-dimensional schematic of the SMR. (**b**) Cross-sectional view showing the layer configuration.

Figure 3 shows a schematic diagram of a typical three-dimensional SMR structure. An alternating current (AC) excitation signal is applied to the top and bottom electrodes via the “Signal” (S) and “Ground” (G) pads, exciting longitudinal bulk acoustic waves in the piezoelectric film. A Bragg reflector layer located beneath the bottom electrode reflects the generated acoustic waves. Research has shown that SMR structures can be divided into two operating modes depending on the thickness of the piezoelectric film: the λ/4-mode and the λ/2-mode (λ represents the resonant wavelength) [22]. It is important to note that using a piezoelectric film in the λ/2-mode has a higher effective electromechanical coupling coefficient than the λ/4-mode, so this study employed the λ/2-mode. In the λ/2-mode configuration, each Bragg reflector layer has a thickness of λ/4, and the piezoelectric layer has a thickness of λ/2. Figure 3a shows a schematic diagram of the λ/2 mode SMR structure used in this study, which consists of three pairs of alternating high and low acoustic impedance layers serving as Bragg reflectors [23]. Figure 3b shows the cross-sectional view structure of the designed SMR, which consists of a piezoelectric film, top/bottom electrodes, and a Bragg reflector stack.

In order to improve the reflectivity of sound waves at the interface, it is necessary to maximize the acoustic impedance mismatch between the two materials as much as possible [24]. Using molybdenum (Mo) and silicon dioxide (SiO_2_) as high acoustic impedance and low acoustic impedance materials, respectively, can effectively improve reflectivity and obtain high *Q* values, which is the basis for achieving high sensitivity of devices in subsequent research. The thicknesses of the Mo and SiO_2_ films are determined using Equation (1).

(1) v=f×4d

where v is the bulk acoustic wave velocity of each film, f is the resonance center frequency, d is the thickness of each film. Given that our target operating frequency is 2.257 GHz, the thickness of the Bragg reflector layer (Mo/SiO_2_) is calculated based on the quarter wavelength condition at the target frequency to maximize the limitation of acoustic energy. The specific thickness calculated is marked in Table 1 below. After determining the material and thickness of the Bragg reflector layer, the selection of the piezoelectric layer is also crucial for device performance. The present study employs AlScN as the piezoelectric layer for the SMR to enhance sensitivity. Nevertheless, the presence of numerous abnormally oriented grains (AOGs) is frequently observed during the deposition of AlScN films via radio frequency (RF) magnetron sputtering. In previous investigation [13,25,26], we used AlN/AlScN composite film to inhibit the grown of AOGs successfully. Consequently, a complete layer stack was designed, comprising an AlN seed layer, Mo electrodes (bottom and top electrodes), an AlN/Al_0.8_Sc_0.2_N composite piezoelectric film, and a Mo/SiO_2_ Bragg reflector. The total thickness required for the resonator can also be calculated using Equation (1), by use the λ/2 mode and a known sound velocity (approximately 11,000 m/s) in AlN/AlScN composite films, with the same target operating frequency of 2.257 GHz. To achieve the target resonance frequency and desired acoustic performance, the thicknesses of Electrode, Piezoelectric and seed layers were also meticulously optimized using a COMSOL (Version 6.1) finite element model. Table 1 summarizes the corresponding thickness of each layer calculated or optimized to achieve the target resonance frequency.

## 3. Fabrication of SMR

**Figure 4:**
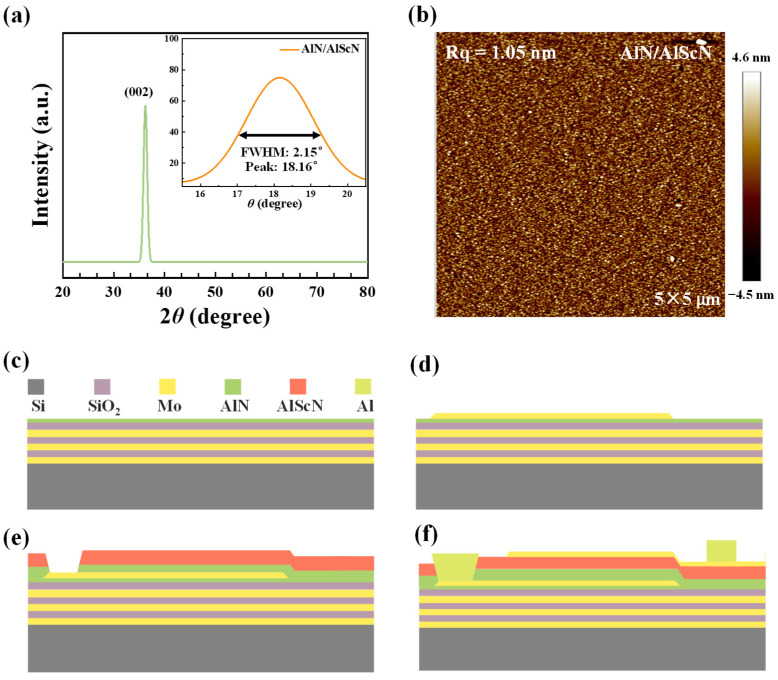
Characterization of the AlN/Al_0.8_Sc_0.2_N composite film and fabrication process of the SMR. (**a**) XRD rocking curves of AlN/Al_0.8_Sc_0.2_N composite film. (**b**) AFM images of AlN/Al_0.8_Sc_0.2_N composite film. (**c**–**f**) Main fabrication process steps of the SMR.

In order to achieve good performance of high sensitivity and high Q value of the device, special attention should be paid to the performance potential of the material, namely the crystallization quality and surface smoothness of the AlN/AlScN composite film in this study. As shown in Figure 4a, the crystalline quality of the AlN/Al_0.8_Sc_0.2_N composite film was characterized by high-resolution X-ray diffractometer (XRD, X’Pert Pro, PANalytical, Almelo, The Netherlands) using ω-scan rocking curve measurements of the (0002) diffraction peak. We overcome the poor crystalline quality of traditional AlScN by employing a heterostructure. The FWHM measured by material characterization was 2.15°, a significant improvement in crystalline quality compared to typical AlScN films (typically 2.5–3.5° [27,28,29]), demonstrating the feasibility of this research approach. However, experimental data indicate that the film width is slightly wider than that of high-quality pure AlN films (0.8–1.5°). This broadening is primarily due to: (i) a lattice mismatch between AlN (α = 3.11 Å) and amorphous SiO_2_, and (ii) a intrinsic lattice distortion caused by Sc substitution in AlN. Despite this slight broadening, its impact is negligible in practical applications. Importantly, the achieved FWHM of 2.15° lies within the range typically required for high-performance piezoelectric devices [27,28], confirming the effectiveness of our composite structure in balancing Sc incorporation and crystal quality. Atomic force microscopy (AFM) revealed no AOGs and a smooth surface morphology with a root-mean-square (RMS) roughness of 1.05 nm (Figure 4b), which contributes to reduced acoustic scattering and an enhanced resonator *Q* factor.

After verifying that our AlScN film met device-level support requirements, we began fabricating the process. The fabrication process flow of the SMR device is depicted in Figure 4c–f. As shown in Figure 4c, Mo and SiO_2_ were alternately deposited as Bragg reflector layers. A 612 nm thick molybdenum (Mo) film was deposited on an 8-inch-high resistivity silicon substrate using radio frequency reactive magnetron sputtering. Subsequently, a 404 nm thick layer of SiO_2_ as grown using Plasma Enhanced Chemical Vapor Deposition (PECVD). By precisely controlling the film thickness and alternately depositing three layers of Mo and SiO_2_ films, a complete Bragg reflector structure is constructed. And after each layer is deposited, the excess SiO_2_ and Mo are ground flat using Chemical Mechanical Polishing (CMP) to flatten the wafer surface, which is beneficial for the subsequent deposition of thin films. Figure 4d shows the preparation of the seed layer and bottom electrode. Firstly, a 25 nm thick AlN seed layer was deposited on the deposited Bragg reflector using low-temperature sputtering process and pulsed DC magnetron sputtering to improve the substrate environment and enhance the growth quality of Mo and AlN thin films [29]. Subsequently, 200 nm of Mo was deposited on the AlN seed layer as the bottom electrode layer, and finally the bottom electrode was patterned and etched. Figure 4e shows the deposition of a 400 nm thick AlN and 400 nm AlScN composite film as the piezoelectric layer using the N_2_ pretreatment process of magnetron sputtering combined with AlN/AlScN composite piezoelectric film used in our previous work [30]. A pre-treatment process was added to the program of the pulsed DC magnetron sputtering machine, which only introduced 50 sccm of N_2_ gas into the chamber, ionized and bombarded the Al-Sc alloy target. Growing AlScN thin films in this pure nitrogen environment is beneficial for reducing defects in AlScN grains. Subsequently, inductively coupled plasma (ICP) etching was used to expose the bottom electrode pads. As shown in Figure 4f, 200 nm of Mo is deposited on the AlN/AlScN piezoelectric layer as the top electrode layer, followed by patterned photolithography and etching of Mo using ICP process to form the top electrode pattern. Figure 4c–f sequentially illustrate the complete fabrication process. Throughout the fabrication process, the piezoelectric film quality was monitored. The deposition was performed at a substrate-target distance of 50 mm and a temperature of 200 °C. Through low-temperature sputtering and precise thickness control technologies, the high-quality thin film of the new composite material in this study was realized as a device, which reduced the acoustic wave loss, greatly improved the electromechanical coupling coefficient, and further enhanced the sensitivity of the entire device.

**Figure 5:**
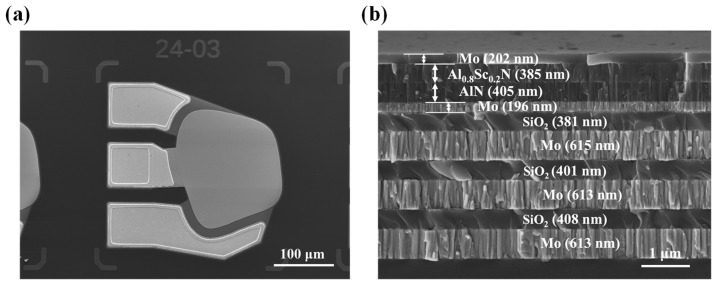
SEM characterization of the fabricated SMR. (**a**) Top-view SEM image of the fabricated SMR. (**b**) Cross-sectional SEM image of the fabricated SMR.

While the complete fabrication process provides a detailed description of each key step and corresponding process parameters, scanning electron microscope (SEM) surface and cross-sectional images offer a better view of the device’s actual structure and morphology, further demonstrating the device’s structural integrity and improved quality at a microscopic level. Figure 5a,b presents the surface and cross-sectional images of the SMR device we fabricated. We used a SEM to carefully examine the device. The results show that the functional layers remain well-formed, and the boundaries between them are clear. Small thickness variations at the nanometer scale appeared during film deposition. This is due to the inherent process characteristics of thin film deposition. The characterization after manufacturing confirms that all functional layers are uniform and have good structural integrity, and small thickness changes do not affect the overall performance of the equipment.

The above discussion on the preparation of AlN/Al_0.8_Sc_0.2_N composite films and their application in SMR devices demonstrates the effective results achieved through the innovative design and preparation methods employed in this study. This specific heterostructure design effectively overcomes the performance instability typical of conventional AlScN films by simultaneously improving crystal quality and piezoelectric response. A precise low-temperature sputtering process and multilayer Bragg reflector design achieve a high-quality factor for device fabrication. SEM and AFM characterization further validates device integrity and stability, ensuring greater assurance for practical application. This composite film not only represents a technological breakthrough but also demonstrates excellent application prospects, providing new ideas and a material and process foundation for the development of highly sensitive, high-frequency, and highly stable SMRs. Further practical applications are anticipated in areas such as wireless communications, microelectromechanical systems, and new energy technologies.

## 4. Experiment and Result

**Figure 6:**
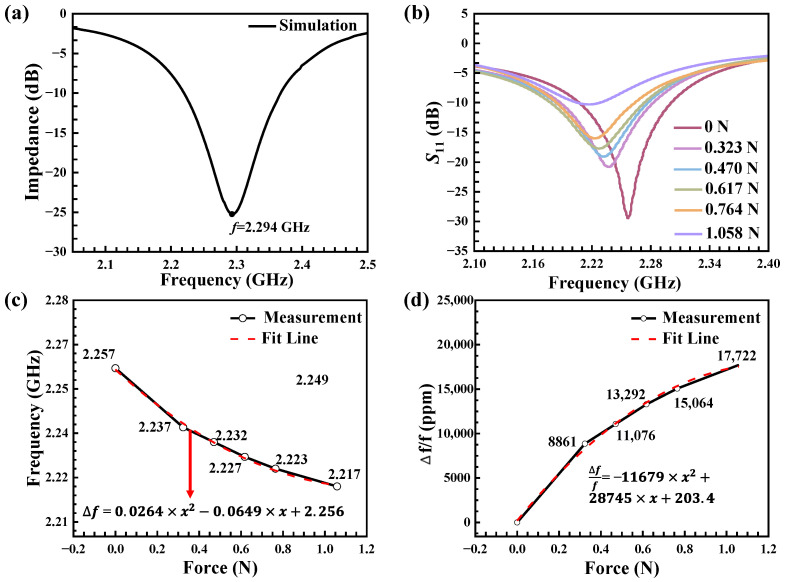
Load sensing performance of the SMR-based sensor. (**a**) Simulated S_11_ response of the SMR. (**b**) S_11_ response under different applied forces. (**c**) Resonance frequency variation as a function of force. (**d**) Force sensitivity of this load sensor.

Before preparing the SMR, we first designed and optimized the layer thickness through finite-element simulation to achieve the target resonance frequency. Figure 6a presents the simulated S_11_ response, which exhibits a clear resonance dip. The series resonance frequency (*f_s_*) is identified at the reflection minimum, approximately 2.294 GHz. A slight discrepancy exists between the simulated (2.294 GHz) and measured (approximately 2.257 GHz) series resonant frequencies. This is attributed to minor deviations between the as-deposited film properties (such as thickness, density, and stiffness) and the idealized parameters used in the simulation model, which is a common occurrence in MEMS fabrication. Crucially, the simulation successfully guided the design to achieve a high-performance resonator at the target frequency range.

In addition to characterizing and analyzing the device materials, testing the overall sensor performance in a practical setup is also essential. We designed and used the lever-probe setup shown in Figure 1a to test the performance of our SMR force sensor. The lever-pin assembly weighs 33 g and applies an initial force of 0.323 N at standard gravity (9.8 m/s^2^). To minimize the influence of contact variations, a fixed polished pin tip and precise micro-positioning alignment were maintained throughout the testing process. The initial force of 0.323 N on the lever component establishes a stable mechanical reference point, and the stable mechanical baseline established relative to the 0.323 N lever preload characterizes all force sensitive responses. By applying a lever ratio (lever arm L1 = 20 cm, load arm L2 = 30 cm, with a factor of 0.667), additional weights of 10 g, 20 g, 30 g, and 50 g were added, resulting in effective masses on the SMR of 48 g, 63 g, 78 g, and 108 g, respectively. This corresponds to forces of 0.47 N, 0.617 N, 0.764 N, and 1.058 N, respectively. The overall uncertainty in the applied force is dominated by the lever arm length measurement (±1%) and mass calibration (±0.5%). These systematic errors were quantified, and their combined effect is significantly smaller than the observed nonlinear force-frequency response, thus not affecting the fundamental conclusions of this study. After the relevant equipment was prepared, the SMR was mounted on a printed circuit board (PCB), and its ground-signal-ground (GSG) pads were connected to a Keysight network analyzer (N5222B, Keysight Technologies, Santa Rosa, CA, USA) via gold wires and subminiature version A (SMA) connectors for test, ensures the accuracy and stability of signal measurement.

The transmission coefficients S_11_ of the SMR under different forces are presented in Figure 6b, and the S_11_ measurements clearly show the frequency shift caused by force. The resonance frequency ($f_{r}$) and quality factor (*Q*) of the device are critical figures of merit. The resonance frequency ($f_{r}$) is identified as the frequency at which the S_11_ response reaches its minimum value (i.e., the dip of the resonance curve). The *Q*, which quantifies the energy loss relative to the energy stored in the resonator, was calculated using the following standard formula based on the 3 dB bandwidth method [31]:

(2) $$Q\;=\frac{f_{r}}{{\Delta f}_{3dB}}$$

where ${\Delta f}_{3dB}$ is the bandwidth of the resonance peak measured at the 3 dB points (i.e., the frequencies where the magnitude of S_11_ is 3 dB higher than the minimum value). Formula 2 is a universal formula that can be used for both *fₛ* and *fₚ*. This method provides a reliable and widely accepted measure of the resonator’s performance. Based on the above calculation method, the *Q* value obtained in this study is 250.

We can observe that the *Q* factor of the resonator gradually decreases with increasing force values in Figure 6b. This is attributed to the lattice distortion in the region contacting the pin, which modifies the local elastic properties and thereby hinders acoustic wave propagation. Furthermore, the acoustic wave is scattered at the contact interface, and its energy is dissipated due to the locally altered boundary conditions, resulting in energy loss. This phenomenon becomes more pronounced with increasing force, causing the *Q* value leading to a further decrease. The data in Figure 6b reflects the sensitive response ability of this SMR sensor device to external forces and shows good signal characteristics.

Figure 6c,d shows the variation in resonance frequency (Δ*f*) of S_11_ as a function of the force in scatter plots, the variable x represents the applied force in units of Newtons (N). There is a quadratic correlation (Δ*f* = *aF*^2^ + *bF* + *c*) between the resonance frequency shift and the applied load. After approximation, the formula is:

(3) $$∆f=0.0264\times x^{2}-0.0649\times x+2.256$$

To verify the stability of the data, the fitting error was obtained in the covariance matrix of the least squares regression: *a* = 0.0264 ± 0.0033; *b* = −0.0649 ± 0.0036; *c* = −6 ± 0.0009. The second-order polynomial was selected because it provides a physically consistent model that captures the fundamental electromechanical response of the piezoelectric film: the linear term (*b*) corresponds to the primary piezoelectric effect, while the quadratic term (*a*) accounts for the nonlinear stress-stiffening that becomes significant under the high localized stress induced by the pin contact. This minimal adequate model not only yields an excellent fit to the experimental data but also defines the precise calibration curve that is responsible for the device’s high sensitivity. Corresponding, the corresponding force sensitivities formula is:

(4) $$\frac{∆f}{f}=-11,679\times x^{2}+28,745\times x+203.4$$

After fitting the error, a = −11,679 ± 1441; b = 28,745 ± 1608; c = 203.4 ± 408.4. For the two relationships depicted in Figure 6c,d, the coefficient of determination (R^2^) reached 0.997, indicating that the model captured over 99.7% of the variability in the experimental data. The excellent quality of the quadratic fit was quantitatively confirmed by the error analysis. The precision of the fitted parameters was also confirmed by their standard errors. The specific parameter precisions are noted in the legends of Figure 6c,d.

Through these two formulas, we established a mathematical analysis model that can achieve a quantitative correspondence between force and frequency shift. Within the analysis range, the SMR-based force sensor achieved an average sensitivity of 37.79 MHz/N (16,744 ppm/N), showing an extremely high force detection capability, far higher than traditional BAW resonators, capable of detecting smaller force changes, and suitable for high-precision force sensing applications. Table 2 summarizes the performance test data of previous pressure sensors based on bulk acoustic wave resonators. By comparing the relevant data of BAW resonator sensors based on Quartz, Si, AlN, AlScN and other materials in existing research results, the results show that the sensitivity of the device in this study far exceeds that of traditional materials and devices. This reflects the significant performance improvement brought about by the optimization of material composite structure and process, shows the broad prospects of the device in this study in the field of micro force sensing.

## 5. Conclusions

This research has successfully designed and experimentally verified a high-sensitivity SMR force sensor based on an AlN/AlScN heterostructure. By using Al_0.8_Sc_0.2_N as the piezoelectric layer, the electromechanical coupling coefficient ($K_{t}^{2}$ ≈ 15%) has been significantly improved at the high resonance frequency of 2.257 GHz, overcoming the sensitivity limitation of using pure AlN as the piezoelectric layer. Compared with traditional force sensors, the high-frequency and high-sensitivity SMR force sensor proposed in this research features the following technological breakthroughs. The AlN/AlScN composite film prevents abnormal grain growth, achieving an XRD half-width of just 2.15° and a surface roughness of only 1.05 nm. This greatly enhances crystal quality and the efficiency of acoustic wave transmission. The λ/2 mode resonator paired with a Mo/SiO_2_ Bragg reflector boosts the electromechanical coupling effect. The lever system focuses stress at the tip, increasing the deformation effect on the piezoelectric material. Beyond these advantages, this sensor is also compact in size, possessing good application and development potential for high-precision static load measurement in emerging fields such as industrial manufacturing, microrobotics, aerospace engineering and other fields.

## Figures and Tables

**Table 1:** The designed thicknesses of resonator dimensions.

Layer	SiO_2_ (Bragg Reflector)	Mo (Bragg Reflector)	AlN (Seed Layer)	Mo (Electrode)	AlN (Piezoelectric)	Al_0.8_Sc_0.2_N
**Thickness**	404 nm	612 nm	25 nm	200 nm	400 nm	400 nm

**Table 2:** Comparison of performance reported in previous works and in this study.

Piezoelectric Film	Sensitivity	Resonator Type	References
Quartz	0.379 MHz/N	Quartz Crystal Microbalance	[32]
Quartz	0.011 MHz/N	Quartz Crystal Microbalance	[33]
Si	0.216 MHz/N	MEMS Resonator	[34]
AlN/Al_0.8_Sc_0.2_N/Si	103.2 ppm/N (0.046 MHz/N)	SAW	[18]
ZnO	32.76 MHz/N	BAW-FBAR	[18]
AlN	9.34 MHz/N (10,614 ppm/N)	BAW-FBAR	[18]
AlN/Al_0.8_Sc_0.2_N/Mo	37.79 MHz/N (16,744 ppm/N)	BAW-SMR	This work

## Data Availability

The data are contained within the article.
